# Advances of multi-omics applications in hepatic precancerous lesions and hepatocellular carcinoma: The role of extracellular vesicles

**DOI:** 10.3389/fmolb.2023.1114594

**Published:** 2023-03-16

**Authors:** Xiaona Lu, Yuyao Li, Yue Li, Xuemei Zhang, Jia Shi, Hai Feng, Yueqiu Gao, Zhuo Yu

**Affiliations:** ^1^ Department of Liver Disease, Shuguang Hospital Affiliated to Shanghai University of Traditional Chinese Medicine, Shanghai, China; ^2^ Institute of Infectious Disease, Shuguang Hospital Affiliated to Shanghai University of Traditional Chinese Medicine, Shanghai, China

**Keywords:** multi-omics, extracellular vesicle, hepatic precancerous lesions, hepatocellular carcinoma, early diagnosis, immunotherapy

## Abstract

Due to the lack of distinct early symptoms and specific biomarkers, most patients with hepatocellular carcinoma (HCC) are usually diagnosed at advanced stages, rendering the treatment ineffective and useless. Therefore, recognition of the malady at precancerous lesions and early stages is particularly important for improving patient outcomes. The interest in extracellular vesicles (EVs) has been growing in recent years with the accumulating knowledge of their multiple cargoes and related multipotent roles in the modulation of immune response and tumor progression. By virtue of the rapid advancement of high-throughput techniques, multiple omics, including genomics/transcriptomics, proteomics, and metabolomics/lipidomics, have been widely integrated to analyze the role of EVs. Comprehensive analysis of multi-omics data will provide useful insights for discovery of new biomarkers and identification of therapeutic targets. Here, we review the attainment of multi-omics analysis to the finding of the potential role of EVs in early diagnosis and the immunotherapy in HCC.

## 1 Introduction

Hepatocellular carcinoma (HCC) is the predominant type of primary liver cancer with comprising 75%–85% of cases, and the third leading cause of cancer-related death worldwide (Sung et al., 2021). The carcinogenesis mostly occurs in liver cirrhosis and is thought to be a complex multistep process (Sciarra et al., 2016). The precancerous stage is characterized with the appearance of dysplastic nodules in cirrhosis, which are divided into low-grade dysplastic nodules (LGDN) and high-grade dysplastic nodules (HGDN) according to the degree of cellular and structural atypia (International Consensus Group for Hepatocellular Neoplasia, 2009). HGDN was a potent predictor of hepatocarcinogenesis, with reported malignant transformation rates of 46.2%, 61.5%, and 80.8% at 1, 3, and 5 years (Borzio et al., 2003; Kobayashi et al., 2006; Sato et al., 2015). Patients with precancerous lesions and early-stage HCC can benefit from curative therapies including hepatic resection, liver transplantation, and radiofrequency ablation, leading to the attainment of 70%–80% of 5-year survival rate (Cho et al., 2011; Villanueva, 2019; Llovet et al., 2021). However, due to the lack of distinct early symptoms and specific biomarkers, most patients are usually diagnosed at advanced stages when the tumor is unresectable, with a median survival of only 6 months (Hollebecque et al., 2015; Hartke et al., 2017). Systemic therapy for advanced HCC has only been limited to anti-angiogenic tyrosine kinase inhibitors (TKIs) until 2017. In recent years, the deep research of immune checkpoint inhibitors (ICIs) favored the development of immune-based systemic therapy in clinical practice. However, immunotherapy has only shown strong antitumor response to some HCC patients, and main challenges are the identification of sensitive population and the optimization of therapeutic strategies (Sangro et al., 2021). Therefore, finding of early diagnostic specific markers and establishment of effective strategies for immunotherapy have attracted tremendous attention.

**FIGURE 1:**
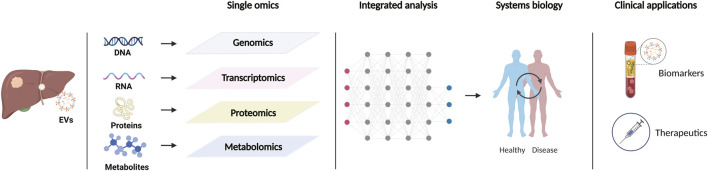
Schematic representation of multi-omics approaches to extracellular vesicles in the study of hepatic precancerous lesions and hepatocellular carcinoma. Created with https://BioRender.com.

An interest in extracellular vesicles (EVs) sharply increased in recent years following the discovery that EVs act as carriers for the intercellular transport of proteins, nucleic acids, lipids, metabolites, etc. EVs are a heterogenous group of lipid bilayer-delimited particles that are naturally released by almost all types of cells and can be broadly sorted into three main classes according to their biosynthesis or secretion process: exosomes, microvesicles/microparticles/ectosomes, and apoptotic bodies (Yáñez-Mó et al., 2015). Exosomes originate from the endosomal system in which endosomes invaginate to form multivesicular bodies (MVBs), and further develop into intraluminal vesicles (ILVs). ILVs are released as “exosomes” upon fusion of MVBs with the plasma membrane. Ectosomes (microparticles/microvesocles) are generated by the outward budding and fission of the plasma membrane, and subsequently released into the extracellular space. Apoptotic bodies are blebs yielded and released by dying cells that undergo apoptosis (Colombo et al., 2014; van Niel et al., 2018). Since no specific markers have been found to identify EV subtypes, “extracellular vesicle” or analogous appellations are the currently preferred nomenclature for these particles (Théry et al., 2018). During the biogenesis, different types of EVs are selectively enriched with a variety of cellular bioactive molecules as cargos. Although some proteins may be shared among EV subtypes, the character of vesicle cargo is highly dependent on the type of donor cells, and their conditions in the physiological or pathological process (van Niel et al., 2018). In the tumor microenvironment, EVs can transfer molecular cargoes among tumor cells, stromal cells, and immune cells for intercellular communication (Becker et al., 2016). Studies have demonstrated the critical role of EVs in tumor progression and antitumor immune responses (Marar et al., 2021). As the properties of EVs as signaling vehicles continue to be revealed, their roles in cancer diagnosis and treatment are gradually emerging. Recent development and broad availability of multi-omics technologies, including genomics, transcriptomics, proteomics, metabolomics, and lipidomics, have promoted a more in-depth study of EVs, which provides profound prospect for the discovery of new candidate biomarkers and the establishment of effective strategies for cancer therapy (Figure 1). In this review, we summarize the progression of multiomics-integrated analysis of EVs for the early diagnosis of precancerous lesions and HCC and discuss a new class of EV-centered cancer immunotherapies.

## 2 Applications of EVs in early diagnosis

EVs are extensively found in different biological fluids, and carry various biomolecules from parental cells, including functional proteins, nucleic acids, and lipids metabolites. They initiate a diversity of physiological and pathological processes in the way of cell-cell communication via delivery of the cargo to recipient cells, and exert the prominent function in many diseases including cancers (Yáñez-Mó et al., 2015; Becker et al., 2016). EVs that are released from tumor cells into the blood can provide a survey of the entire tumor and represent as biomarkers that can be collected and analyzed from blood samples, offering great potential for early diagnosis of cancer. However, there is currently no single optimal separation method to obtain pure EVs, so the choice depends on the downstream application and the scientific question to be solved (Théry et al., 2018).

### 2.1 Genomics/transcriptomics

Recent studies have demonstrated the presence of complete genomic DNA (gDNA), mitochondrial DNA (mtDNA), and even viral DNA in EVs(Cai et al., 2013; Thakur et al., 2014; Sansone et al., 2017; Lázaro-Ibáñez et al., 2019; Saari et al., 2020; Liu H. et al., 2022). Bioactive DNA-carrying EVs play crucial roles in maintaining cellular homeostasis, regulating immune responses, and participating in tumor formation and progression (Takahashi et al., 2017; Torralba et al., 2018; Malkin and Bratman, 2020; Elzanowska et al., 2021; Hagey et al., 2021). It has been found that oxidized mtDNA-enriched EVs can synergize with acetaldehyde to activate oxidative stress and multiple oncogenic pathways in cancerous cells for the promotion of alcohol-related liver cancer development (Seo et al., 2019). Data analysis based on the examination of next-generation sequencing (NGS) technique showed that EV mtDNA covered the entire mitochondrial genome, in which mtDNA end sites, cleavage numbers, and copy numbers were significantly reduced in EVs from HCC patients compared with healthy controls, indicating the potential value of EV mtDNA signatures in the diagnosis of HCC(Li Y. et al., 2020). Although their biological significance has been recognized, EV DNAs are still less explored, and their role in precancerous lesions and HCC still needs to be further verified.

EVs are also rich in various types of RNAs, which exhibit great potential for early detection of HCC. A study containing 291 participants revealed that mRNA levels of serum exosomal heterogeneous ribonucleoprotein H1 (hnRNPH1) were significantly higher in HCC than in cirrhosis, chronic hepatitis B, and healthy controls (Xu et al., 2018b). The *hnRPH1* gene encodes a splicing regulator that can stimulate pre-mRNA cleavage and polyadenylation, and is abnormally expressed in a number of human cancers such as esophageal cancer, pancreatic cancer, colon cancer, and prostate cancer (Honoré et al., 2004; Sun et al., 2016; Yang et al., 2016). Here exosomal *hnRNPH1* mRNA was identified as an effective marker for HBV-related HCC evidenced by an AUC of 0.865 (95% CI = 0.808–0.922, *p* = 0.003), and its combination with AFP could further improve the diagnostic validity. Lactate dehydrogenase C (LDHC) is a germ cell-specific member of the lactate dehydrogenase family. LDHC activation in cancers may provide a metabolic rescue pathway in tumor cells by using lactate for ATP delivery. Studies have confirmed that *LDHC* mRNA is expressed in various tumor tissues such as lung cancer, melanoma, breast cancer, and kidney cancer (Koslowski et al., 2002; Wang et al., 2018a). Recently, Cui Z *et al.* found that the expression of *LDHC* mRNA was also significantly upregulated in serum EVs of HCC patients, which could clearly distinguish early-stage HCC patients from healthy controls (Cui et al., 2020). Compared to total circulating EVs, tumor-derived EVs have more potential to be used as disease-specific biomarkers. However, current conventional isolation techniques based on EVs’ physical properties fail to isolate tumor-specific EVs. To overcome this issue, Sun N *et al.* have developed a covalent chemistry-based HCC-specific EV purification system, EV Click Chips, which enables the isolation of plasma HCC-derived EVs in high efficiency and purity (Sun et al., 2020). Moreover, by coupling EV Click Chips with a downstream reverse-transcription droplet digital PCR assay, 10 well-validated HCC-specific mRNA transcripts, such as alpha-fetoprotein (AFP), glypican 3 (GPC3), albumin (ALB), apolipoprotein H (APOH), were selectively quantified for the calculation of a digital score. The resulting score in EVs exhibited great potential for non-invasive detection of early-staged HCC in high-risk cirrhosis patients, evidenced as an area under the ROC curve (AUC) of 0.93 (95% CI, 0.86 to 1.00; sensitivity = 94.4%, specificity = 88.5%).

In addition to mRNA, some non-coding RNAs (ncRNAs), including microRNAs (miRNAs), long non-coding RNAs (lncRNAs), circular RNAs (circRNAs), transfer RNA (tRNA), have also exhibited instrumental impact on HCC progression. MiRNAs are a class of endogenous small ncRNAs with the length of 19–25 nucleotides, which serve as critical regulators of post-transcriptional gene expression and play an important role in cell growth, differentiation, development, and apoptosis (Saliminejad et al., 2019). In recent years, a large number of researches have shown that many miRNAs can act as tumor suppressors or oncogenes to promote the occurrence and development of tumors (Di Leva et al., 2014). One of the representative paradigm is miR-21 that was early discovered to be widespread in human cells or tissues. Increasing studies have manifested that miR-21 functions as an oncogene to involve the post-transcriptional gene regulation, cell differentiation, proliferation and apoptosis, which are closely related to tumorigenesis (Singh et al., 2021). Furthermore, miR-21 was evidenced to be enriched in serum EVs of patients with liver diseases, and the level of EV miR-21 in HCC patients is significantly higher than in patients with chronic hepatitis B, liver cirrhosis or healthy donors, indicating the potential of miR21-containing EVs as a diagnostic marker for HCC(Wang H. et al., 2014; Pu et al., 2018; Mjelle et al., 2019; Sorop et al., 2020; Wang et al., 2020). In addition, other circulating miRNAs, such as miR-221/222, miR-224, miR-155, may also play an important role in the early stage of hepatocarcinogenesis (Wang et al., 2009; Zhang Y. et al., 2012; Cui et al., 2019). Analysis of miRNA expression in selected serum EVs showed that miR-18a, -221, -222, and -224 were specifically upregulated, while miR-101, -106b, - 122 and -195 were downregulated in HCC patients (Sohn et al., 2015). Among them, miR-221 was evidenced to promote hepatcarcinogenesis by dysregulating DNA damage-inducible transcript 4 (DDIT4), the modulator of mTOR pathway, whereas miR-101 that is downregulated in HCC may induce apoptosis and inhibit tumorigenicity via targeting Mcl-1 (Su et al., 2009; Pineau et al., 2010). Combining transcriptome techniques plus existing datasets from GEO and TCGA databases, Ghosh S *et al.* analyzed the miRNA profile of HBV- and HCV-infected HCC versus normal or adjacent tissues, and Panther/Gene Ontology enrichment/Cytoscape analysis indicated that the targets were mostly involved in carcinogenesis pathways (Ghosh et al., 2020). After validated to be in plasma-derived exosomes from HBV/HCV-infected non-HCC, such as chronic hepatitis and cirrhosis, and HCC samples, four miRNAs including miR-10b-5p, miR-221-3p, miR-223-3p and miR-21-5p were identified as novel potential biomarkers for early HCC diagnosis, and their combination showed better sensitivity in distinguishing HCC, especially with low AFP expression. Similarly, the study by Cho HJ et al. confirmed that miR-10b-5p in serum EVs is a promising diagnostic biomarker for early-stage HCC with the AUC equal to 0.934 (sensitivity = 90.7%, specificity = 75.0%) (Cho et al., 2020b). In another study, analysis of plasma miRNA microarray was performed to screen the miRNA with different expression in EVs derived from HCC, cirrhosis and healthy control. As a result, three miRNAs including miRNA-26a, miRNA-29c and miRNA-199a were identified to be significantly reduced in HCC-derived EVs. Further analysis indicated that these miRNAs may act as tumor suppressors in HCC, and the examination of EVs with the miRNAs-embedded panel showed higher accuracy in the diagnosis of HCC(Lin and Zhang, 2019; Yang J. et al., 2022). In addition, other numerous EVs miRNAs have been identified as potential early-diagnosed markers for HCC, including but not limited to miR-34a, miR-93, miR-101, miR-122, miR-125b, miR -148a, miR-182, miR-301a, miR-320d, miR-373, miR-483-5p, miR-665, miR-3129, miRNA-4661-5p, miR-4746-5p, and the combinations of multiple miRNAs would present higher diagnostic utility (Qu et al., 2017; Wang et al., 2018b; Xue et al., 2018; Xue et al., 2019; Cho et al., 2020a; Li W. et al., 2020; Muhammad Yusuf et al., 2020; Sheng et al., 2020; Sun et al., 2021; Yang et al., 2021; Chen S. et al., 2022; Deng et al., 2022; Lin et al., 2022). These markers are expected to construct new liquid biopsy modalities in the near future.

Recently, many lncRNAs and circRNAs derived from EVs have been proposed as potential candidate biomarkers for HCC diagnosis. LncRNAs are ncRNAs with a length of more than 200 nucleotides, which play an important role in regulating the transcriptome level. They were found to be differentially expressed in tumors and were directly associated with the transformation of normal cells into tumor cells (Huarte, 2015). By virtue of TCGA_LIHC and Catholic_LIHC projects (two human HCC whole transcriptome datasets), Kim SS *et al.* identified specific tumor-driving lncRNA candidates in serum EVs by comparing the expression of lncRNA in HCC and non-tumor tissues. In the end, the EV lncRNA-MALAT1, -DLEU2, -HOTTIP, -SNHG1, and -LINC00853 were identified as promising diagnostic markers for early-stage HCC, even in AFP-negative patients (Kim et al., 2020; Kim et al., 2021). MALAT1 is a representative onco-lncRNA that stimulates tumor growth and metastasis through multiple mechanisms in different tissues (Wang J. et al., 2014; Hou et al., 2017). DLEU2, HOTTIP, and SNHG1 have also been reported as onco-lncRNAs in HCC, while LINC00853 is currently poorly understood for the role in cancer and deserved for further study. RNA-seq technology coupled with multi-stage validation revealed many meaningful lncRNAs candidates with different expression, including lncRNA-THEMIS2-211, -RP11-85G21.1, -ENSG00000248932.1, -ENST00000440688.1, -ENST00000457302.2, -ENSG00000258332.1, and -LINC00635(Xu et al., 2018a; Huang et al., 2020; Lu et al., 2020; Yao et al., 2022). These lncRNAs are packed in EVs and play an instrumental role in inducing HCC formation and development. Their expression levels in EVs of HCC patients were significantly higher than those of chronic hepatitis/cirrhosis or healthy controls, presenting a potent diagnostic ability, and even better than AFP in diagnosing early-stage HCC. Other studies further added EV LINC00161, CRNDE, lnc-FAM72D-3, and lnc-EPC1-4 as potential biomarker candidates for HCC diagnosis (Sun et al., 2018; Yao et al., 2020; Wang T. et al., 2021). Moreover, lncRNA-HEIH showed specific diagnostic values in HCV-related HCC patients (Zhang et al., 2018). CircRNAs are a class of endogenous non-coding RNAs with a covalent closed loop structure, which are enriched and stable in EVs (Li et al., 2015; Kristensen et al., 2019). Hu K *et al.* found elevated levels of circCMTM3 in exosomes from HCC patients. Further experiments showed that exosomal circCMTM3 could promote angiogenesis through the miR-3619-5p/SOX9 axis, thereby promoting HCC tumorigenesis (Hu et al., 2021). In contrast, exosome-delivered circ_0051443 could inhibit the malignant biological behavior of HCC cells by inducing apoptosis and arresting cell cycle, and its expression in plasma EVs of HCC patients was significantly lower than that of healthy controls, evidenced by an AUC of 0.8089 (Chen et al., 2020). Another study showed that circ_0070396 was highly expressed in EVs of HCC and outperformed AFP in distinguishing HCC patients from chronic hepatitis B/cirrhosis or healthy donors (Lyu et al., 2021). Through transcriptome sequencing analysis and large sample clinical validation, circ_0006602 and circ_0028861 in EVs have been identified as candidate biomarkers for HCC, which can be used for early diagnosis and screening of HCC(Wang Y. et al., 2021; Guo et al., 2021). Functional experiments displayed that exo_circ_0006602 could significantly improve the proliferation, migration, and invasion of HCC cells, with the promoted expression of tumor proliferation-related protein. Exosomal circ_0028861 may affect HCC progression by regulating the miRNA targets and downstream tumor-related signaling pathways. Besides, other study reported the role of EVs tRNA-derived small RNA (tsRNA, a novel small non-coding RNA) in the diagnosis of HCC. It was found that tsRNAs are ubiquitous in exosomes, and the levels of four tsRNAs (tRNA-ValTAC-3, tRNA-GlyTCC-5, tRNA-ValAAC-5 and tRNA-GluCTC-5) were significantly elevated in plasma exosomes of HCC patients compared with healthy controls, revealing the diagnostic value of tsRNA in HCC (Zhu et al., 2019).

Comprehensive analysis of EV RNAs is a viable option in the diagnosis of patients with early-staged HCC (Wu Z. H. et al., 2022). Li Y *et al.* found that the sequencing profile of extracellular vesicle long RNA in blood (exLR, including mRNA, lncRNA, and circRNA) can distinguish cancer patients from healthy individuals, and in this way revealed that 8 exLRs had the potential as HCC diagnostic biomarkers in a multistage cohort (Li et al., 2019). The exLR-based HCC classifier has utility beyond AFP, not only for diagnosing early-stage or AFP-negative HCC, but also for distinguishing HCC patients from those with hepatitis, cirrhosis, or benign tumors. Another study evaluating a largely unexplored EVs-associated unannotated small RNA clusters (smRCs) demonstrated that the 3-smRC signature was significantly overexpressed in plasma EVs from HCC patients, which exhibited high sensitivity and specificity for the detection of early HCC in an independent validation cohort evidenced by the AUC of 0.87 (von Felden et al., 2021). Through a series of studies, Abd El Gwad A et al. demonstrated the role of three EV RNA-based biomarkers (lncRNA-RP11-513I15.6, miR-1262 and RAB11A) in inducing the formation of precancerous lesions in rats, and their excellent ability to differentiate HCC patients from chronic hepatitis and healthy volunteers (Abd El Gwad et al., 2018; Matboli et al., 2019; Hasanin et al., 2020). Comprehensive analysis of other extracellular RNAs also revealed the diagnostic value of different RNA detection panels in HCC, such as the combination of circRNA (SNORD3B-1, circ-0080695) with miR-122 (Zhu et al., 2021; Cabiati et al., 2022). Biomarkers identified by genomics/transcriptomics are summarized in Table 1. With the rapid development of liquid biopsy technology, circulating EV miRNAs, lncRNAs, and circRNAs may become more reliable biomarkers for HCC prediction in the future.

**TABLE 1:** Potential biomarkers identified in genomics or transcriptomics studies.

Biomarker	Molecule type	Vesicle type	Sample	Control group	Number	AUC	SEN	SPE	Refs
mtDNA	DNA	EVs	Plasma	CHB and healthy controls	HCC (*n* = 15), CHB (*n* = 5), Healthy (*n* = 5)	NR	NR	NR	Li et al. (2020b)
10 HCC-specific mRNA^a^	mRNA	EVs	Plasma	LC	HCC (*n* = 36), LC (*n* = 26)	0.93	94.4%	88.5%	Sun et al. (2020)
*hnRNPH1*	mRNA	Exosomes	Serum	CHB	HCC (*n* = 88), CHB (*n* = 68)	0.865	85.2%	76.5%	Xu et al. (2018b)
*LDHC*	mRNA	Exosomes	Serum	Healthy controls	HCC (*n* = 102), Healthy (*n* = 100)	0.9451	88.2%	93.3%	Cui et al. (2020)
miR-122, miR-21, miR-96	miRNA	Exosomes	Plasma	LC	HCC (*n* = 50), LC (*n* = 50)	0.924	82%	92%	Wang et al. (2020)
				Healthy controls	HCC (*n* = 50), Healthy (*n* = 50)	0.996	96%	98%	Wang et al. (2020)
miR-21-5p, miR-92a-3p	miRNA	Exosomes	Plasma	LC and healthy controls	HCC (*n* = 48), LC (*n* = 38), Healthy (*n* = 20)	0.85	NR	NR	Sorop et al. (2020)
miR-21, miR-144	miRNA	EVs	Serum	CHB and healthy controls	HCC (*n* = 24), CHB (*n* = 16), Healthy (*n* = 17)	0.78	NR	NR	Pu et al. (2018)
miR-21	miRNA	Exosomes	Serum	CHB and healthy controls	HCC (*n* = 30), CHB (*n* = 30), Healthy (*n* = 30)	NR	NR	NR	Wang et al. (2014a)
miR-224	miRNA	Exosomes	Serum	Healthy controls	HCC (*n* = 89), Healthy (*n* = 50)	0.91	NR	NR	Cui et al. (2019)
miR-18a, -221, -222, -224 and miR-101, -106b, -122, -195	miRNA	Exosomes	Serum	CHB and LC	HCC (*n* = 20), CHB (*n* = 20), LC (*n* = 20)	NR	NR	NR	Sohn et al. (2015)
miR-10b-5p, miR-221-3p, miR-223-3p, miR-21-5p	miRNA	Exosomes	Plasma	CH	HCC (*n* = 38), CH (*n* = 35)	0.86	74%	86%	Ghosh et al. (2020)
				CH and LC	HCC (*n* = 38), CH (*n* = 35), LC (*n* = 25)	0.8	58%	95%	Ghosh et al. (2020)
miR-10b-5p, miR-221-3p, miR-223-3p	miRNA	Exosomes	Plasma	CH	HCC (*n* = 38), CH (*n* = 35)	0.84	86%	66%	Ghosh et al. (2020)
				CH and LC	HCC (*n* = 38), CH (*n* = 35), LC (*n* = 25)	0.74	86%	53%	Ghosh et al. (2020)
miR-10b-5p	miRNA	Exosomes	Serum	CHB, LC and healthy controls	HCC (*n* = 90), CHB (*n* = 27), LC (*n* = 33), Healthy (*n* = 28)	0.934	90.7%	75.0%	Cho et al. (2020b)
miR-26a, miR-29c, miR-199a	miRNA	Exosomes	Plasma	LC	HCC (*n* = 50), LC (*n* = 50)	0.965	92%	90%	Yang et al. (2022a)
				Healthy controls	HCC (*n* = 50), Healthy (*n* = 50)	0.994	100%	96%	Yang et al. (2022a)
miR-26a	miRNA	Exosomes	Serum	CHB	HCC (*n* = 30), CHB (*n* = 30)	0.922	NR	NR	Lin and Zhang (2019)
miR-29c	miRNA	Exosomes	Serum	CHB	HCC (*n* = 30), CHB (*n* = 30)	0.934	NR	NR	Lin and Zhang (2019)
miR-21	miRNA	Exosomes	Serum	CHB	HCC (*n* = 30), CHB (*n* = 30)	0.935	NR	NR	Lin and Zhang (2019)
miR-122, 148a	miRNA	Exosomes	Serum	Healthy controls	HCC (*n* = 59), Healthy (*n* = 31)	NR	NR	NR	Deng et al. (2022)
miR-34a	miRNA	Exosomes	Serum	Healthy controls	HCC (*n* = 60), Healthy (*n* = 60)	0.664 ± 0.0499	78.3%	51.7%	Chen et al. (2022a)
miR-483-5p	miRNA	Exosomes	Plasma	LC and healthy controls	HCC (*n* = 60), LC (*n* = 47), Healthy (*n* = 52)	0.898	85%	90.38%	Lin et al. (2022)
miR-101, miR-125b	miRNA	Exosomes	Serum	Healthy controls	HCC (*n* = 20), Healthy (*n* = 20)	0.953	NR	NR	Sun et al. (2021)
miR-320d	miRNA	Exosomes	Serum	Healthy controls	HCC (*n* = 110), Healthy (*n* = 40)	0.8694	NR	NR	Li et al. (2020a)
miR-4661-5p, miR-4746-5p	miRNA	Exosomes	Serum	CH, LC and healthy controls	HCC (*n* = 72), CH (*n* = 22), LC (*n* = 28), Healthy (*n* = 22)	0.947	81.8%	91.7%	Cho et al. (2020a)
miR-182, miR-301a, miR-373	miRNA	Exosomes	Serum	LC	HCC (*n* = 26), LC (*n* = 26)	NR	NR	NR	Muhammad Yusuf et al. (2020)
miR-93	miRNA	Exosomes	Serum	Healthy controls	HCC (*n* = 85), Healthy (*n* = 23)	0.825	NR	NR	Xue et al. (2018)
miR-665	miRNA	Exosomes	Serum	Healthy controls	HCC (*n* = 30), Healthy (*n* = 10)	NR	NR	NR	Qu et al. (2017)
miR-628-3p, miR-941, miR-584-5p, miR-106-3p	miRNA	Exosomes	Serum	CHB and LC	HCC (*n* = 51), CHB (*n* = 18), LC (*n* = 20)	NR	94.1%	68.4%	Sheng et al. (2020)
miR-122, miR-125b, miR-145, miR-192, miR-194, miR-29a, miR-17-5p, miR-106a	miRNA	Exosomes	Serum	Healthy controls	HCC (*n* = 80), Healthy (*n* = 30)	0.535 to 0.850	NR	NR	Xue et al. (2019)
miR-148a	miRNA	Exosomes	Serum	LC	HCC (*n* = 50), LC (*n* = 40)	0.891	NR	NR	Wang et al. (2018b)
miR-122	miRNA	Exosomes	Serum	Healthy controls	HCC (*n* = 50), Healthy (*n* = 50)	0.99	100%	92.0%	Wang et al. (2018b)
MALAT1	lncRNA	sEVs	Serum	CH, LC and healthy controls	HCC (*n* = 72), CH (*n* = 21), LC (*n* = 25), Healthy (*n* = 21)	0.908	92.063%	81.579%	Kim et al. (2021)
DLEU2	lncRNA	sEVs	Serum	CH, LC and healthy controls	HCC (*n* = 72), CH (*n* = 21), LC (*n* = 25), Healthy (*n* = 21)	0.882	80.263%	82.540%	Kim et al. (2021)
HOTTIP	lncRNA	sEVs	Serum	CH, LC and healthy controls	HCC (*n* = 72), CH (*n* = 21), LC (*n* = 25), Healthy (*n* = 21)	0.879	82.253%	77.465%	Kim et al. (2021)
SNHG1	lncRNA	sEVs	Serum	CH, LC and healthy controls	HCC (*n* = 72), CH (*n* = 21), LC (*n* = 25), Healthy (*n* = 21)	0.898	80.769%	85.246%	Kim et al. (2021)
LINC00853	lncRNA	sEVs	Serum	CH, LC and healthy controls	HCC (*n* = 90), CH (*n* = 28), LC (*n* = 35), Healthy (*n* = 29)	0.935	83.33%	89.77%	Kim et al. (2021)
THEMIS2-211	lncRNA	Exosomes	Plasma	Healthy controls	HCC (*n* = 97), Healthy (*n* = 101)	0.818	82.8%	70.8%	Yao et al. (2022)
RP11-85G21.1 (lnc85)	lncRNA	Exosomes	Plasma	LC and healthy controls	HCC (*n* = 112), LC (*n* = 43), Healthy (*n* = 52)	0.873	80.0%	74.5%	Huang et al. (2020)
ENSG00000248932.1, ENST00000440688.1, ENST00000457302.2	lncRNA	Exosomes	Plasma	Healthy controls	HCC (*n* = 180), Healthy (*n* = 180)	0.838	NR	NR	Lu et al. (2020)
				CH	HCC (*n* = 180), CH (*n* = 180)	0.534	NR	NR	Lu et al. (2020)
ENSG00000258332.1	lncRNA	Exosomes	Serum	CHB	HCC (*n* = 55), CHB (*n* = 60)	0.718	73.5%	80.5%	Xu et al. (2018a)
LINC00635	lncRNA	Exosomes	Serum	CHB	HCC (*n* = 55), CHB (*n* = 60)	0.731	79.6%	75.2%	Xu et al. (2018a)
LINC00161	lncRNA	Exosomes	Serum	Healthy controls	HCC (*n* = 56), Healthy (*n* = 56)	0.794	75.0%	73.2%	Sun et al. (2018)
CRNDE	lncRNA	Exosomes	Serum	Healthy controls	HCC (*n* = 166), Healthy (*n* = 100)	0.839	69.3%	85.0%	Wang et al. (2021a)
lnc-FAM72D-3	lncRNA	Exosomes	Serum	Healthy controls	HCC (*n* = 45), Healthy (*n* = 45)	0.584	NR	NR	Yao et al. (2020)
lnc-EPC1-4	lncRNA	Exosomes	Serum	Healthy controls	HCC (*n* = 45), Healthy (*n* = 45)	0.576	NR	NR	Yao et al. (2020)
circ_0051443	circRNA	Exosomes	Plasma	Healthy controls	HCC (*n* = 60), Healthy (*n* = 60)	0.8089	NR	NR	Chen et al. (2020)
circ_0070396	circRNA	Exosomes	Plasma	Healthy controls	HCC (*n* = 111), Healthy (*n* = 54)	0.8574	62.16%	98.15%	Lyu et al. (2021)
				CHB	HCC (*n* = 111), CHB (*n* = 50)	0.7741	76.58%	68%	Lyu et al. (2021)
				LC	HCC (*n* = 111), LC (*n* = 58)	0.6609	46.85%	81.03%	Lyu et al. (2021)
circ_0006602	circRNA	Exosomes	Plasma	Healthy controls	HCC (*n* = 87), Healthy (*n* = 30)	0.9564	93.33%	89.74%	Guo et al. (2021)
circ_0028861	circRNA	Exosomes	Serum	CHB and LC	HCC (*n* = 56), CHB (*n* = 57), LC (*n* = 47)	0.79	67.86%	82.69%	Wang et al. (2021b)
tRNA-ValTAC-3, tRNA-GlyTCC-5, tRNA-ValAAC-5, tRNA-GluCTC-5	tsRNA	Exosomes	Plasma	Healthy controls	HCC (*n* = 5), Healthy (*n* = 5)	NR	NR	NR	Zhu et al. (2019)
8 exLRs	long RNA	EVs	Plasma	Hepatic benign disorders and Healthy controls	HCC (*n* = 33), Hepatic benign disorders (n = 6), Healthy (*n* = 33)	0.9627	85%	95%	Li et al. (2019)
3-smRC (smRC119591, smRC135709, smRC48615)	small RNA	EVs	Plasma	Chronic liver disease and non-CLD	HCC (*n* = 105), Chronic liver disease (*n* = 85), non-CLD (*n* = 19)	0.87	86%	91%	von Felden et al. (2021)
lncRNA-RP11-513I15	lncRNA	Exosomes	Serum	CHC and healthy controls	HCC (*n* = 60), CHC (*n* = 42), Healthy (*n* = 18)	0.963	96.7%	95%	Abd El Gwad et al. (2018)
miR-1262	miRNA	Exosomes	Serum	CHC and healthy controls	HCC (*n* = 60), CHC (*n* = 42), Healthy (*n* = 18)	0.847	95%	80%	Abd El Gwad et al. (2018)
RAB11A	mRNA	Exosomes	Serum	CHC and healthy controls	HCC (*n* = 60), CHC (*n* = 42), Healthy (*n* = 18)	0.822	75%	73.3%	Abd El Gwad et al. (2018)
circRNA (SNORD3B-1, circ-0080695), miR-122	long RNA	Exosomes	Plasma	Healthy controls	HCC (*n* = 38), Healthy (*n* = 37)	0.894	NR	NR	Zhu et al. (2021)

^a^ 10 mRNA, alpha-fetoprotein (AFP), glypican 3 (GPC3), albumin (ALB), apolipoprotein H (APOH), fatty acid binding protein 1 (FABP1), fibrinogen beta chain (FGB), fibrinogen gamma chain (FGG), alpha 2-HS glycoprotein (AHSG), retinol binding protein 4 (RBP4), and transferrin (TF).

CH, chronic hepatitis; CHB, chronic hepatitis B; CHC, chronic hepatitis C; LC, liver cirrhosis; HCC, Hepatocellular carcinoma; NR, not reported; mtDNA, mitochondrial DNA; mRNA, messenger RNA; miRNA, microRNA; lncRNA, long non-coding RNA; circRNA, circular RNA; tsRNA, tRNA-derived small RNA; exLR, extracellular vesicle long RNA; SEN, sensitivity; SPE, specificity.

### 2.2 Proteomics

Besides nucleic acids, proteins are also critical cargoes in EVs. Proteins packed and transferred via EVs play a vital role in the progression of HCC(Wang et al., 2017; Fu et al., 2018; Jiang et al., 2020; Liu et al., 2021; Tey et al., 2022). Polymeric immunoglobulin receptor (pIgR) and galectin-3-binding protein (LG3BP) are both oncogenic proteins that promote cellular proliferation, transformation and invasion in HCC progression (Serizawa et al., 2015; Yue et al., 2017). EVs have been evidenced to be enriched with pIgR that can drive stemness-like conversion and tumorigenesis by activating PDK1/Akt/GSK3β/β-catenin signaling axis (Tey et al., 2022). In addition, pIgR and LG3BP were found to be significantly overexpressed in circulating EVs of HCC patients and exhibited higher diagnostic capacity than AFP(Arbelaiz et al., 2017). Pyruvate kinase M2 isoform (PKM2), an enzyme that plays a key role in the glucose metabolism of cancer cells, induces macrophage differentiation and remodels the tumor microenvironment through EV excretion, which level is positively correlated with hepatocarcinogenesis (Hou et al., 2020). The testing of clinical samples also validated a significant upregulation of plasma EV PKM2 levels from HCC patients. Sun N *et al.* developed and optimized an HCC-specific EV surface protein detection method (referred as HCC EV ECG score), based on three HCC EV subpopulations of EpCAM^+^CD63^+^, CD147^+^CD63^+^, and GPC3^+^ CD63^+^, by which EVs were isolated from peripheral blood, and HCC EV surface proteins were detected. This new established detection model was confirmed to have an accuracy of more than 93% (AUC >0.93) in distinguishing early HCC from cirrhosis with excellent detection performance (Sun et al., 2022). LC3B (MAP1LC3B) is a widely used marker of autophagy. Recent studies revealed that LC3B^+^ EVs carrying HSP90α are the master regulators of tumor progression, and the level of plasma HSP90α^+^ LC3B^+^ EVs in HCC patients were significantly higher than those in non-cancerous controls, suggesting the potential role as a diagnostic marker for HCC(Chen Y. Q. et al., 2022). Julich-Haertel H *et al.* isolated and detected various tumor-associated EV populations from patient serum and plasma via Fluorescence-activated cell scanning (FACS), and found that AnnexinV^+^ EpCAM^+^ ASGPR1^+^ EV and AnnV^+^ CD44v6^+^ EV were excellent in distinguishing liver malignancies from cirrhotic/control subjects (Julich-Haertel et al., 2017; Urban et al., 2020). These data suggest that the EV phenotype can serve as a novel HCC liquid biopsy biomarker.

To overcome the disadvantages of most traditional EV testing regarding huge samples, tedium, time consuming, low purity, and high cost, as well as the technical barriers of EV protein molecular analysis, some novel strategies for optimizing EV isolation, detection, and analysis have been developed (Di et al., 2020; Wu et al., 2020; Yang K. et al., 2022). Yang K *et al.* developed a new affinity method to isolate EVs by adhering highly abundant phosphatidylserine molecules on the surface of EVs to immobilized small peptide molecules (SiO2 microspheres), which can recover complete EVs in a short time with high yield and low cost (Yang K. et al., 2022). By comparing the biological information of differentially expressed proteins in serum EVs isolated by this method, early HCC patients and healthy individuals can be well differentiated. Another study also reported a nanozyme-assisted immunosorbent assay (NAISA), a platform based on the installation of 2 nm gold nanoparticles onto the phospholipid membrane of EVs, which enables rapid, sensitive, and specific analysis of EV proteins (Di et al., 2020). Protein expression in cell-derived EVs was measured by NAISA, and the results indicated that GPC-3 was specifically upregulated in HCC cell-derived EVs. GPC-3 is a member of the glypican family involved in the regulation of cell proliferation and apoptosis, which highly expresses as the indicator of malignant transformation of hepatocytes, and may mediate oncogenesis and oncogenic signaling pathways (Yao et al., 2016). Researchers further validated in clinical samples that both EV GPC-3 and CEA could effectively discriminate HCC patients from chronic hepatitis B patients and healthy controls, indicating the enormous potential of these EV proteins as effective biomarkers for early diagnosis of HCC. The study by Aydin Y *et al.* also confirmed the role of GPC3-positive circulating EVs in the detection of HCC in patients with liver cirrhosis (Aydin et al., 2021).

The nature of relatively high throughput in proteomic analysis shows great advantage in screening potential diagnostic biomarkers for HCC. Through differential proteomic analysis, multiple EV proteins were identified as potential candidate biomarkers and arrayed in testing panels, such as “LBP, KV311 and CO9,” “VWF, LGALS3BP, TGFB1, SERPINC1, HPX, HP, HBA1, FGA, FGG and FGB” and “Cofilin-1 and CCT8” (Cho et al., 2021; Zhao et al., 2021; Ye et al., 2022). These proteins play important roles in regulating cell growth, proliferation, differentiation, and innate and adaptive immune responses. Another study also showed that annexin A2 (ANXA2) and versican core protein (VCAN) were elevated in plasma EVs in HCV-related HCC patients, which can be used to characterize HCV-induced HCC(Taleb et al., 2017). ANXA2 belongs to the calcium-dependent phospholipid-binding protein family and plays an important role in the malignant transformation of HCC(Mohammad et al., 2008). VCAN, an extracellular matrix component, has been shown to also be involved in fibrotic deposition in the liver, which may limit its applicability as a diagnostic marker for HCC in the setting of cirrhosis. Biomarkers identified by proteomics are summarized in Table 2.

**TABLE 2:** Potential biomarkers identified in proteomics studies.

Biomarker	Vesicle type	Sample	Control group	Number	AUC	SEN	SPE	Refs
PIGR	EVs	Serum	Healthy controls	HCC (*n* = 29), Healthy (*n* = 32)	0.837	82.8%	71.8%	Arbelaiz et al. (2017)
LG3BP	EVs	Serum	Healthy controls	HCC (*n* = 29), Healthy (*n* = 32)	0.904	96.9%	71.8%	Arbelaiz et al. (2017)
PKM2	Ectosomes	Plasma	Healthy controls	HCC (*n* = 6), Healthy (*n* = 6)	NR	NR	NR	Hou et al. (2020)
HCC EV ECG score^a^	EVs	Plasma	LC	HCC (*n* = 35), LC (*n* = 37)	0.93	91%	81%	Sun et al. (2022)
HSP90α, LC3B	EVs	Plasma	Non-malignant liver disease and healthy controls	HCC (*n* = 51), Non-malignant liver disease (*n* = 33), Healthy (*n* = 30)	0.9595	86.00%	96.67%	Chen et al. (2022b)
AnnexinV, EpCAM, ASGPR1	Microparticles	Serum	LC	HCC (*n* = 86), LC (*n* = 49)	0.7322	81.40%	46.94%	Julich-Haertel et al. (2017)
AnnexinV, CD44v6	EVs	Serum	Biliary cancer	HCC (*n* = 29), Biliary cancer (*n* = 60)	0.81	91.7%	69.0%	Urban et al. (2020)
370 differentially-expressed proteins	Exosomes	Serum	Intrahepatic cholangiocarcinoma and healthy controls	HCC (*n* = 9), Intrahepatic cholangiocarcinoma (*n* = 9), Healthy (*n* = 9)	NR	NR	NR	Yang et al. (2022b)
GPC-3	Exosomes	Serum	CHB and healthy controls	HCC (*n* = 12), CHB (*n* = 12), Healthy (*n* = 6)	NR	NR	NR	Di et al. (2020)
CD63	Exosomes	Serum	Healthy controls	HCC (*n* = 25), Healthy (*n* = 25)	0.95	NR	NR	Aydin et al. (2021)
			LC	HCC (*n* = 25), LC (*n* = 25)	0.87	NR	NR	Aydin et al. (2021)
GPC-3	Exosomes	Serum	Healthy controls	HCC (*n* = 25), Healthy (*n* = 25)	1.0	NR	NR	Aydin et al. (2021)
			LC	HCC (*n* = 25), LC (*n* = 25)	0.95	NR	NR	Aydin et al. (2021)
CO9	Exosomes	Plasma	Healthy controls	HCC (*n* = 7), Healthy (*n* = 4)	0.929	85.7%	100.0%	Ye et al. (2022)
			CHB	HCC (*n* = 7), CHB (*n* = 10)	0.857	85.7%	90%%	Ye et al. (2022)
LBP	Exosomes	Plasma	Healthy controls	HCC (*n* = 7), Healthy (*n* = 4)	0.964	100.0%	75.0%	Ye et al. (2022)
KV311	Exosomes	Plasma	LC	HCC (*n* = 7), LC (*n* = 7)	0.857	85.7%	85.7%	Ye et al. (2022)
VWF, TGFB1, LGALS3BP, SERPINC1, HPX, HP, HBA1, FGA, FGG, FGB	Exosomes	Serum	Healthy controls	HCC (*n* = 20), Healthy (*n* = 10)	NR	NR	NR	Zhao et al. (2021)
CCT8, Cofilin-1	Exosomes	Serum	CHB, LC and healthy controls	HCC (*n* = 132), CHB (*n* = 25), LC (*n* = 33), Healthy (*n* = 34)	0.829	85.61%	61.96%	Cho et al. (2021)
ANXA2, VCAN	Microparticles	Plasma	LC and healthy controls	HCC (*n* = 16), LC (*n* = 22), Healthy (*n* = 18)	NR	NR	NR	Taleb et al. (2017)

^a^ Accounted from EpCAM^+^ CD63^+^ HCC EVs, CD147^+^ CD63^+^ HCC EVs, and GPC3^+^ CD63^+^ HCC EVs.

CHB, chronic hepatitis B; LC, liver cirrhosis; HCC, Hepatocellular carcinoma; NR, not reported; SEN, sensitivity; SPE, specificity.

### 2.3 Metabolomics/lipidomics

Like nucleic acids and proteins, metabolites are also an important type of molecules carried by EVs. The metabolome of EVs mainly includes lipids, amino acids, organic acids, carnitine, steroids, vitamins, sugars and their complexes, as well as nucleotides and nucleosides (Zebrowska et al., 2019; Luo et al., 2020). In cancers, these molecular metabolites could be transferred to parenchymal cells and their surrounding stromal cells through the EV pathway, and influence the metabolism of recipient cells (Puhka et al., 2017). Metabolic reprogramming has been shown to be a hallmark of cancer, and researchers have recently increasingly focused on characterizing signatures of altered metabolism in cancer. The emerging role of EV-mediated metabolic reprogramming in tumor microenvironment remodeling and cancer progression has gradually emerged (Yang et al., 2020). Liu et al. (2021) observed reduced levels of triosephosphate isomerase 1 (TPI1), a glycolytic enzyme, in EVs released from HCC cells enhanced aerobic glycolysis-driven hepatocarcinogenesis. Metabolites represent downstream products of genes, transcripts, and proteins in multiple biological reactions, providing a more comprehensive molecular perspective and thus more likely to reveal dynamic changes in biological state (Luo et al., 2018; Tian et al., 2018). However, studies involving the intact metabolites present in EVs are scarce, and the reported metabolic analyses of EVs have mainly focused on lipids so far. Lipids are not only involved in building the structure of EV membranes, but also play an important role in the formation and release of EVs into the extracellular environment (Skotland et al., 2019). With the development of lipidomic analysis methods, the identification of novel lipid-based biomarkers has become available in practice. Sanchez JI et al. analyzed the difference of lipids in plasma EVs from 31 HCC patients and 41 cirrhotic patients using ultra-high resolution mass spectrometry (for untargeted lipidomic analysis), and discovered that ten classes of lipids were significantly enriched in EVs from HCC patients compared with non-HCC ones, while four classes were depleted (Sanchez et al., 2021). Logistic regression analysis revealed the close association of HCC with the enriched lipids, including sphingosine (SPH), disolysin (DLCL), lysophosphatidylserine (LysoPS), (O-acyl)-1-hydroxy fatty acids (OAHFA), and the depleted ones, such as gangliosides (GD1a) and fatty acids (FA). The difference in the composition and abundance of lipids could facilitate to distinguish HCC from non-HCC patients (*p* < 0.001), suggesting that the lipidomic signature of EVs has the potential to be a candidate biomarker for early detection of HCC in the future.

However, due to the paucity of current knowledge about lipid metabolism and the defects of current corresponding technologies, the research of metabolomics/lipidomics is far from as sufficient as that of proteomics and transcriptomics, and its clinical application is still limited. Therefore, the EVs metabolome remains to be digged more deeply and extensively.

### 2.4 Integromics and systems biology

#### 2.4.1 Multi-omics combined

Single omics analyses can provide information on biological processes that differs in different life configurations, or on disease conditions compared to normal ones. However, these analyses can only provide limited insights, not suggesting comprehensive information on interactions among DNA, RNA, proteins, and metabolites. The most important question is whether the markers will eventually have the power to test efficacy in clinical practice. A single marker seems difficult to meet this requirement. Therefore, the combination of multi-omics is another promising direction to discover early cancer diagnostic markers. The integrated analysis of data obtained by different omics approaches is expected to provide new insights into complex biological systems and reveal the interaction network between all molecular-level processes. The multi-omics analysis of EVs is to use a variety of high-throughput technologies, including genomics, transcriptomics, proteomics, metabolomics, etc., to comprehensively analyze various cargo molecules in EVs from multiple aspects, in order to understand their biological function and clinical application prospect. The advantage of EV multi-omics analysis is that by analyzing the composition of various active molecules in EV, the origin, biological function and mechanism of action of EV can be more accurately determined, thereby revealing its role in physiology and disease, and providing a theoretical basis for clinical diagnosis and treatment. Meanwhile, EV multi-omics analysis is characterized by high throughput, high sensitivity, high specificity, and non-invasiveness, allowing simultaneous analysis of multiple types of molecules, thus improving research efficiency and accuracy. Integrative omics analysis of EVs in the context of hepatic precancerous lesions and hepatocellular carcinoma has been seldom reported, but multi-omics studies with applications to other cancers are continuously emerging, especially in the area of biomarker discovery. Using a combination of proteomics, metabolomics, and lipidomics, Eylem et al. (2020) characterized colorectal cancer (CRC)-derived exosomes and identified signature molecules that could differentiate CRC patients from healthy individuals. Luo et al. (2021) dissected the transcriptome and proteome profiles of exosomes released from human lung adenocarcinoma stem-like cells (LSLC) and found that several LSLC markers were highly expressed and associated with poor survival, which may serve as promising liquid biopsy biomarkers for the diagnosis of lung adenocarcinoma. Given the biodiversity and heterogeneity of EVs, such integrative multi-omics data analysis can be used to reveal the multi-level biological data of EVs to explore the functions of EVs more deeply and accurately.

However, multi-omics analysis of EVs also faces some challenges. First, the purification and quantification of EVs are still problematic, thus, it remains a difficult task to extract pure EVs from complex body fluids accurately and efficiently. Secondly, the molecular composition of EVs is heterogeneous and complex, and how identifying and classifying different types of EVs and their molecular compositions is a difficult problem. In addition, EV multi-omics analysis requires the analysis and integration of a large amount of data, so how to effectively integrate data and perform bioinformatics analysis to systematically and comprehensively annotate complex biological network regulation is also a challenge.

#### 2.4.2 Systems biology

Systems biology, as a rookie in the post-genomic era, is becoming a more sophisticated approach to integromices. This approach, characterized by the integration of systems theory and experimental and computational methods, combines experimental data at multiple molecular levels with computational models and treats the system as a whole, providing a new perspective for revealing the role of EVs in human health and disease (Gézsi et al., 2019; Çelebier and Ercan, 2021). The integrated analysis of the interactions of different molecules in the genome, transcriptome, proteome, metabolome, etc., helps to identify the best biomarker candidates. With the development of multi-omics diagnostic tools, and of new computational methods for data integration, the combination of EVs with multi-omics for the diagnosis of early HCC will illuminate a profound understanding and progress in the future.

## 3 Applications of EVs to immunotherapy

Since the immunoregulatory potential of EVs was first discovered in 1998, novel EVs-centered cancer immunotherapy has been extensively explored (Zitvogel et al., 1998). Recently, EVs have been proposed as a cell-free therapeutic strategy that has attracted considerable attention in the field of cancer immunotherapy (Liu C. et al., 2022). The involvement of EVs in the crosstalk between tumor cells and immune cells in the microenvironment provides many opportunities for cancer immunotherapy. Macrophages are one of the prominent immune cells in the tumor microenvironment and can be polarized to assume different phenotypes under different microenvironment (Lewis and Pollard, 2006). There are two main subgroups designated M1 (classically activated macrophages) and M2 (alternatively activated macrophages) (Mosser and Edwards, 2008). Tumor-associated macrophages (TAMs) are mainly of the M2 phenotype, which play an important role in tumor progression and immunosuppression (Galdiero et al., 2013). Studies have shown that tumor cell-derived EVs can target neighboring macrophages and modulate their conversion to M2 (Li X. et al., 2018); in turn, M2 macrophage-derived EVs can facilitate tumor progression and CD8^+^ T cell exhaustion in HCC mice (Chen J. et al., 2021; Pu et al., 2021). Several engineered EV therapeutic candidates targeting macrophages have been demonstrated to reprogram TAMs to a pro-inflammatory M1 phenotype and enhance immunity against HCC, resulting in marked inhibition of tumor growth (Chen H. et al., 2021; Gunassekaran et al., 2021; Kamerkar et al., 2022).

Immune cell-derived EVs also display the potential for HCC immunotherapy. It was reported that dendritic cell (DC)-derived EVs (DEX) could elicit strong antigen-specific antitumor immune responses and significant tumor regression in HCC mice after intravenous administration (Zuo et al., 2022). Some previous studies also showed that EVs from tumor antigen-expressing DCs can reshape the tumor immune microenvironment, resulting in significant tumor growth retardation and survival prolongation in HCC mice (Lu et al., 2017; Li J. et al., 2018). In addition, pulsed stimulation with tumor cell-derived EVs (TEX) carrying multiple HCC antigens can potentiate DCs immunogenicity and improve vaccines efficiency (Rao et al., 2016; Zuo et al., 2020). Another study also suggested that DEX could induce an equivalent antitumor immune-enhancing effect to DC when combined with microwave ablation (Zhong et al., 2020). These results indicate that DEX has a great application prospect as a cell-free tumor vaccine or a natural antitumor adjuvant, opening up a new avenue for HCC immunotherapy. Moreover, EVs derived from other immune cells such as natural killer cells (NK) have also shown specific tumor-killing effects on HCC cells (Kim et al., 2022); Wu J. et al. (2022) have developed a motion bioreactor that can produce NK EVs on a large scale, which provides technical support for promoting the clinical translation of EVs. The cytotoxic effect of immune cell-derived EVs on tumors greatly enriches the role of EVs in cell-free immunotherapy. With the understanding of the mechanisms of these phenomena, more advances will be elucidated.

Another potential usage of EVs in the HCC immunotherapy is to enhance the efficacy of immune checkpoint inhibitors (ICIs). Systemic therapy based on ICIs has gradually been the mainstay of HCC immunotherapy, but only about 20% of patients show effective response to the treatment. Recent studies have demonstrated that specific types of TEX could promote the immune escape of HCC by inducing the expansion of regulatory T cells and regulatory B cells (Ye et al., 2018; Huang et al., 2022). Wei et al. (2021)found that the *HMGB1*-*RICTOR* crosstalk networks epigenetically impede the response to anti-PD-L1 immunotherapy by upregulating the activity of PD-L1^+^ exosomes in HCC. In addition to T cells and B cells, TEX can also inhibit various innate immune cells, such as NK cells and macrophages. Studies have indicated that specific types of TEX could inhibit the function of NK cells by inhibiting the secretion of IFN-γ and TNF-α in NK cells, and reduce the proportion of NK cells and the tumor infiltration, thus leading to the resistance to anti-PD1 therapy in HCC (Zhang P. F. et al., 2020). Other studies have also found that HCC-derived EVs promote immunosuppression and resistance to anti-PD1 treatment through the elevation of CD39 expression, while inhibition of the ATP-adenosine pathway by targeting CD39 on macrophages can rescue anti-PD1 therapy in HCC (Lu et al., 2021). These findings provide potential therapeutic strategies for HCC patients. Besides, a recent study demonstrated that PD-L1 secreted by TEXs can inhibit T cell activation in draining lymph nodes and promote tumor growth in an immune-dependent manner; whereas blocking EV PD-L1 suppresses tumor growth and overcome resistance to anti-PD-L1 antibodies (Poggio et al., 2019). In addition, altering the function of TEXs is able to downregulate the expression of PD-L1 on macrophages and attenuate the immunosuppressive state, providing a way to improve the efficacy of immunotherapy in HCC patients (Cheng et al., 2017). These studies suggested that immune checkpoint blockade based on EVs is a promising strategy for cancer immunotherapy.

Apart from as carriers to transport various molecules, EVs could act as the barriers to protect the content in a relatively stable milieu by isolating the inside from the outside. Oncolytic virus is a class of natural viruses, which has been genetically reconstructed to function as the tumor vaccine via specifically infecting and killing tumor cells (Zhang K. J. et al., 2012). However, owing to possessing strong immunogenicity, oncolytic virus usually triggers vehement self-attacking immune response to eliminate themselves. To avoid this situation, Zhang Y. et al. (2020) proposed a new strategy for using EVs to pack the oncolytic adenoviruses, and the results showed that EVs-mimetic encapsulation technology can increase virus infection and shield them from neutralizing antibodies in the serum, thus significantly improving the efficacy of oncolytic virotherapy. In another study, EVs was reformed to deliver small interfering RNA specific for β-catenin with the protection from the degradation, which could effectively strengthen the therapeutic response of HCC to anti-PD-1antibodies (Matsuda et al., 2019). In summary, EVs can exert an immunotherapeutic effect through one or more strategies, which provides a new idea for HCC immunotherapy.

Although EVs-based immunotherapy has shown great potential in preclinical studies, there are no reports of its application in the immunotherapy of HCC patients so far. Therefore, there is still a long way to go in implementing it into routine clinical practice. Current tentative applications of extracellular vesicles in clinical HCC immunotherapies were summarized in Table 3.

**TABLE 3:** Clinical applications of extracellular vesicles in HCC immunotherapies.

Type of treatment	Extracellular vesicles	Target	Role	Refs
Engineered exosomes	PIONs@E6	Macrophage	Promotes the polarization of M1 macrophages to enhance their immunity against HCC	Chen et al. (2021a)
Engineered exosomes	IL4R-Exo (si/mi)	Macrophage	Reprograms TAMs into M1-like macrophages and increases antitumor immunity	Gunassekaran et al. (2021)
Engineered exosomes	exoASO-STAT6	Macrophage	Selectively silences STAT6 expression in TAMs and reprograms them to a pro-inflammatory M1 phenotype	Zuo et al. (2022)
DC vaccines	DEX	DC	Promotes DC recruitment and activation to induce tumor-specific immune responses	Kamerkar et al. (2022)
DC vaccines	DEX_AFP_	CD8^+^ T cells	Activate CD8^+^ T lymphocytes to elicit a strong antigen-specific immune response	Lu et al. (2017)
DC vaccines	DC-Dex	T cell	Stimulates naive T cell proliferation and induces T cell activation into antigen-specific cytotoxic T lymphocytes	Li et al. (2018a)
DC vaccines	TEX-N1ND	DC	Enhances the ability of DCs to activate T cells and improves vaccine efficiency	Zuo et al. (2020)
DC vaccines	TEXs	DC	Carries HCC antigens, triggers strong DC-mediated immune response	Rao et al. (2016)
DC vaccines	DEX	CD8^+^ T cells and Treg cells	Increases the number of CD8^+^ T cells and decreases the number of Treg cells	Zhong et al. (2020)
Oncolytic Viro	EVM/VSV-G Ad5-P	Not specified	Enhanced viral infection efficiency, oncolytic ability, and soluble PD-1 production	Zhang et al. (2020b)
ICIs	PD-L1	Not specified	Exosomal PD-L1 gene blockade promotes T cell activity in draining lymph nodes, thereby inducing systemic antitumor immunity and memory	Poggio et al. (2019)
ICIs	HCC-derived exosomes	Macrophage	Exosomes derived from HCC cells treated with 0.1 mM melatonin can downregulate the expression of PD-L1 on macrophages	Cheng et al. (2017)
ICIs	EV-siRNA	HCC cells	Targeting β-catenin to enhance anti-PD-1 therapeutic response	Matsuda et al. (2019)
Others	NK-exo	HCC cells	Inhibition of serine/threonine kinase pathway-related HCC cell proliferation and promotion of caspase activation pathway-related HCC cell apoptosis	Kim et al. (2022)
Others	ADMSC-derived exosomes	NKT-cell	Promote NKT cell anti-tumor immunity	Ko et al. (2015)
Others	Irradiated tumor cells derived-sEVs	DC	Promotes the release and presentation of tumor antigens	Lin et al. (2020)

TAMs, tumor-associated macrophages; DC, dendritic cell; DEX, DC-derived exosomes; DEX_AFP_, exosomes derived from AFP-expressing DCs; DC-Dex, exosomes derived from recombinant adeno-associated viral vector (rAAV)-carrying AFP -transfected DC; TEXs, Tumor cell-derived exosomes; Treg cells, regulatory T cells; TEX-N1ND, TEXs painted with the functional domain of high mobility group nucleosome-binding protein 1 (HMGN1); EVM/VSV-G Ad5-P, extracellular vesicles-mimetic encapsulated a recombinant adenovirus expressing the extracellular domain of PD1; NK-exo, natural killer cell-derived exosomes; ADMSC, adipose-derived mesenchymal stem cells; NKT-cell, natural killer T-cell.

## 4 Conclusion

A growing body of research has provided valuable knowledge and understanding of the role of EVs in HCC. Although EVs hold great promise as potential early diagnostic biomarkers as well as novel cancer immunotherapeutic targets, their use in clinical settings first requires methodological standardization. It has been recognized that EV-based studies are particularly challenging because of their complex biological origins and enormous heterogeneity in size, composition, and source (van Niel et al., 2018). The advancement of high-throughput omics technology provides a good perspective for deciphering the molecular cargo of EVs and enables the collection and integration of data and information at different molecular levels. The information obtained through multi-omics techniques will provide new opportunities for the diagnosis and treatment of HCC and will improve the prognosis of HCC patients. Although further experimental confirmation and clinical validations are required for findings made through emerging omics approaches, we believe these approaches will transform medical practice in the near future. In the future, the implementation of various omics-based technologies and the integration of systems biology methods for the development of EV-based HCC diagnostics will become a promising trend. On the other hand, although significant progress has been made in the application of EVs in the immunotherapy of HCC, it is still in a secondary position in clinical practice at present. Therefore, future work should simultaneously focus on the rational design of engineered EVs in immune regulation and expand their application in cancer immunotherapy.

In addition, it is a little regrettable that although hepatic precancerous lesion was identified clinically more than 10 years ago, the underlying molecular mechanisms involved in its progression and transformation remain unclear, and little is known about the role that EVs play in it. With the advent of the “omics era” and the deepening understanding of EVs, we expect more research on hepatic precancerous lesions and tumorigenesis to provide new strategies for early diagnosis and intervention of disease.
